# Assessment of MgO, ZnO, chitosan hydroxyapatite and silver hydroxyapatite nanoparticles against carbapenem-resistant Gram-negative Egyptian clinical isolates: a combined *in-silico* and *in-vitro* study

**DOI:** 10.1186/s12866-025-04364-y

**Published:** 2025-10-06

**Authors:** Salma Jamal Ahmed, Shima Mahmoud Ali, Mohammed A. Khedr, Mohamed Emara, Selwan Hamed

**Affiliations:** 1 Department of Microbiology and Immunology, Faculty of Pharmacy, Helwan University − Ain Helwan, Helwan, 11795 Egypt; 2 Department of Pharmaceutical Chemistry, Faculty of Pharmacy, Helwan University − Ain Helwan, Helwan, 11795 Egypt

**Keywords:** Nanoparticles, Docking, Carbapenem, Gyrase, Cytotoxicity, Vitamin D

## Abstract

**Background:**

Currently, antimicrobial resistance (AMR) is one of the major global threats to public health; therefore, treating infectious diseases becamemore challenging. In Egypt, the prevalence of carbapenem-resistant Gram-negative bacteria has notably increased, presenting substantial challenges to infection control and therapeutic strategies. New treatment approaches including nanoparticles have been discovered to combat antibiotic resistance.

**Objective:**

We evaluated the potential of nanoparticles as next-generation antimicrobials through a dual approach combining computational modeling and antimicrobial testing of different nanoparticles (NPs); magnesium oxide (MgO NPs), zinc oxide (ZnO NPs), chitosan hydroxyapatite (ChHap NPs) and silver hydroxyapatite (AgHap NPs) against standard strains and clinical bacterial isolates.

**Results:**

The Minimum inhibitory concentration(MIC) showed that AgHap NPs exhibited MIC value of 1.875 mg. mL^-1^ against all the tested bacterial isolates, while MgO NPs exhibited MIC values of 1.5, 1.5, 0.75 and 0.375 mg/mL against *E. coli*,* Klebsiella pneumoniae*, *Pseudomonas aeruginosa* and *Acinetobacter baumannii*, respectively. The cytotoxicity profiles of the nanoparticles were assessed using the human hepatocellular carcinoma cell line (HepG2), demonstrating approximately 40–85% cell viability at concentrations near MIC values of the AgHap NPs and MgO NPs. A molecular docking study revealed that all tested NPs have affinity to gyrase enzyme in*E*.*coli*,transmission electron microscope images (TEM) also revealed a significant disruption of the bacterial cell wall integrity, indicating a potential mechanism of action.

**Conclusion:**

Collectively, MgO NPs and AgHap NPs are promising alternative therapeutic agents for the treatment of resistant Gram-negative clinical infections.

**Graphical abstract:**

Schematic representation of the dual approach used in this study: chemical synthesis of various nanoparticles (MgO, ZnO, ChHap, AgHap), followed by computational analysis and *in- vitro *antimicrobial testing against carbapenem-resistant Gram-negative bacterial strains (CRGNB).

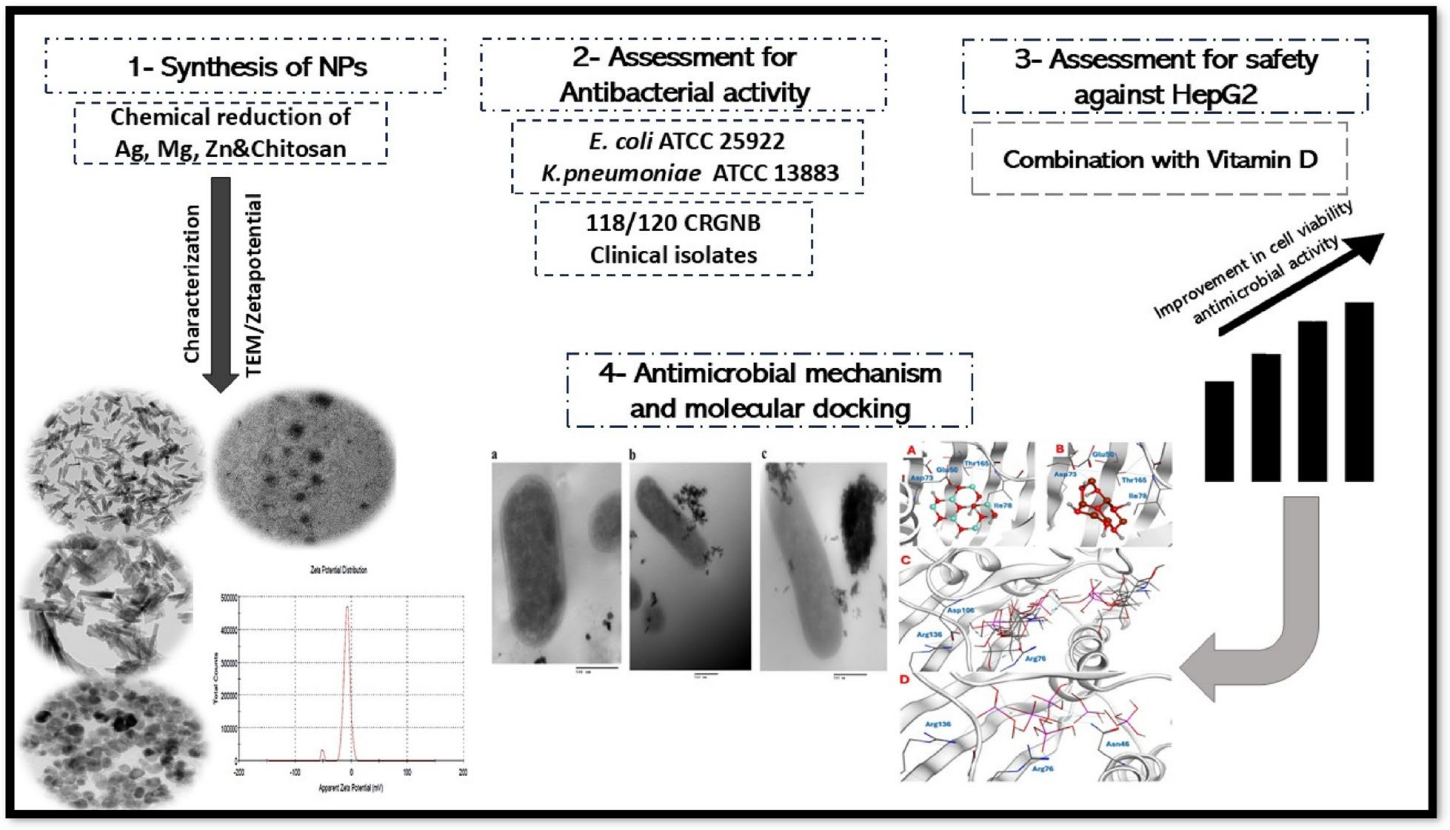

**Supplementary Information:**

The online version contains supplementary material available at 10.1186/s12866-025-04364-y.

## Introduction

Nowadays, antimicrobial resistance (AMR) is one of the ten most serious global threats to public health [1, 2], the World Health Organization (WHO) predicts that by 2050, worldwide deaths due to AMR may exceed 10 million per year [2] as the spread of AMR poses a significant threat to morbidity and mortality worldwide.

In 2024, WHO recognized carbapenem-resistant *A. baumannii*, carbapenem-resistant *P. aeruginosa*, and carbapenem-resistant Enterobacteriaceae as the priority for research and development to have effective antibiotics invented against them, because ofthe high mortality rate of their infections [3].

In Egypt, recent studies have documented a worrisome escalation in the incidence of carbapenem-resistant Enterobacteriaceae (CRE), particularly in tertiary care hospitals [4]. For instance, a study analyzing Gram-negative bacteria isolated from hospitalized patients in Egypt in 2014 found a high level of carbapenem resistance, with 50.8% of the isolates harboring at least one carbapenem resistance gene [5].

Clinicians encounter many difficulties when treatinginfections caused by carbapenem-resistant Gram-negative bacteria, within this context, the usage of drugs like colistin or tigecycline were proved to be associated with unclear effectiveness and nephrotoxicity [6].

The alarming rise in multidrug-resistant (MDR) Gram-negative pathogens, such as *E. coli*, *K. pneumoniae*, *E. aerogenes*, *Citrobacter* spp., *P. mirabilis*, *P. aeruginosa*, and *A. baumannii*, has emerged as a global health threat that significantly limiting the effectiveness of conventional antibiotics. These pathogens employ diverse resistance mechanisms including β-lactamase production, efflux pumps, porin mutations, and biofilm formation, which collectively reduce antibiotic efficacy and complicate treatment strategies. Given the limited success of current therapies, nanotechnology offers promising alternatives by enabling the development of nanoparticles with targeted and multifaceted antimicrobial actions [7]. Nanomaterials such as silver hydroxyapatite (AgHap), chitosan hydroxyapatite (ChHap), zinc oxide (ZnO) [8], and magnesium oxide (MgO) nanoparticles have demonstrated strong bactericidal properties through mechanisms like oxidative stress induction, membrane disruption, and intracellular penetration. Recent studies have emphasized the importance of nanoparticle surface characteristics, size, and composition in enhancing their antibacterial efficacy, especially against MDR strains.

Despite the limitations of conventional antimicrobial treatments, no new class of antibiotics has been developed in the last 20 years; as there hasn’t been much progress in the creation of new medications lately due to financial and economic burdens [9].

Therefore, there are several revolutionary alternative approaches against these infectious MDR bacteria. Among these approaches: nanoparticles, phage therapy, antimicrobial peptides, monoclonal antibodies, anti-virulent therapy and quorum sensing inhibitors seem to have potential promising values [10–12].

Nanoparticles (NPs)exhibit dual strategies to combat AMR including intrinsic bactericidal activity and antibiotic nanocarriers. Moreover, they endure multi-target mechanism of action against bacteria. Furthermore, NPs are classified into two main groups: organic nanoparticles like(ferritin, liposomes, polymers and micelles) and inorganic nanoparticles like (metal nanoparticles and metal oxide nanoparticles) [12, 13].

Despite growing interest in biogenic nanoparticle synthesis, few studies have systematically investigated the comparative antimicrobial performance of multiple nanoparticle types against a broad panel of clinically relevant, carbapenem-resistant isolates. Of the previous types we chose to work on the metal NPs especially AgHap NPs, polymeric NPs as ChHap NPs and metal oxide NPs such as MgO NPs and ZnO NPs because they have several advantages [14–16]. They do not attach to a particular receptor in bacterial cells; consequently, they exhibit bacterial toxicity through non-specific mechanisms.Ultimately, they get a broad range of antibacterial action and hinder the development of bacterial resistance [17].

Magnesium oxide NPs (MgO NPs) possess unique potential to be broad spectrum antimicrobial agent due to their biocompatible and biodegradable properties [18, 19]. Furthermore, MgO NPs exhibit significant antibacterial properties, making them ideal candidates for coatings of medical devices and implants.

Additionally, MgO NPs havealso been recognized by the United States Food and Drug Administration (FDA) organization as harmless material. Consequently, MgO NPs were incorporated in various applications including medical implants and dental composites [19]. On the other hand, AgHap NPs is also a promising path for the development of novel biomedical applications due to its antibacterial activity and biocompatibility [20].

Zinc oxide NPs (ZnO NPs) are also recognized as harmless material by the FDA and are included in numerous applications such as the composition of pharmaceutical drugs, cosmetics, medical implant coatings, medical dressing, drug delivery, sanitizers, food packaging processes, wastewater treatment, and communication [21]. Nevertheless, ChHap NPs lately gathered exceptional attention due to its antibiofilm, broad antimicrobial activity against both Gram-negative and Gram-positive bacteria, low production cost, biocompatibility, abundance in nature, non-toxic and biodegradability, making it a part of many applications such as food packaging, wastewater treatment and biomedical applications [22, 23].

NPs antibacterial mechanism is not fully understood, but it is believed to be mainly due to reactive oxygen species (ROS)production, oxidative stress, etc [10, 24, 25]. Molecular docking has emerged as a powerful computational tool for predicting and visualizing the interaction between NPs and microbial target proteins at the molecular level. By simulating the binding affinity and orientation of NPs or their surface functional groups to essential bacterial proteins, docking studies can provide valuable insights into potential mechanisms of antimicrobial action [26]. Recent studies have employed molecular docking as a powerfulin silico tool to predict the antibacterial potential of NPsbysimulating possible interactions with key bacterial targets [27] enabling a more comprehensive understanding of how engineered nanoparticles exert their effects against MDR pathogens, including carbapenem-resistant Gram-negative bacteria(CRGNB) [28].

Nevertheless, NPs induce cytotoxicity in humans due to their increased ability to permeate tissues and form aggregates because of its small size [29]. Based on previous studies, it was found that the addition of an adjunct antioxidant such as N-acetyl cysteine or Vitamin D could improve NPs cell viability [30, 31].

Therefore, the present study aims to bridge this gap by evaluating the antimicrobial potential of AgHap, ChHap, ZnO, and MgO nanoparticles through a dual strategy involving molecular docking and in-vitro testing, to provide a deeper understanding of their therapeutic applicability against resistant Gram-negative pathogens.In this study, we used a unique dual approach that integrates computational molecular docking with experimental antimicrobial testing to evaluate the efficacy of diverse NPs including MgO, ZnO, ChHap, and AgHap against CRGNB. This also included investigating the proposed mechanism of action and studying the cytotoxic effect of the most promising NPs and their synergism with Vitamin D.

## Materials and methods

### Experimental design

In the current study, the prepared metal (silver hydroxyapatite nanoparticles (AgHap NPs)), polymeric(chitosan hydroxyapatite nanoparticles (ChHap NPs))and metal oxide(magnesium oxide nanoparticles (MgONPs) and zinc oxide nanoparticles (ZnO NPs)) were characterized by their size and shape. Then, the antibacterial activity of eachtype nanoparticles (NPs) against some standard bacterial strains and carbapenem-resistant Gram-negative clinical isolates was tested using broth microdilution technique. Cytotoxicitywas determined by the most promising nanoparticles at various concentrations; Vitamin D was used as an adjunct to improve cell viability. In addition to, the effect of those NPson the bacterialcell wall integrity was determined using Transmission electron microscope(TEM) imaging at sub-lethal concentrations.

### Preparation of nanoparticles

The NPs were synthesized via a chemical reduction route. Magnesium oxide nanoparticles (MgO NPs) were produced by Nanotech, Cairo, using a hydrothermal technique. A magnesium precursor solution (0.1 mM, 1.8 g) was dissolved in 100 mL deionized water, and sodium hydroxide (0.1 M NaOH) was introduced to induce precipitation. The formation of a white suspension signified nucleation of MgO NPs. The mixture was magnetically stirred for 60 min, then transferred into a Teflonlined stainlesssteel autoclave and heated at 150 °C for 24 h. After cooling, the precipitate was washed with deionized water and ethanol repeatedly until achieving neutral pH (~ 7). The product was then centrifuged at 4000 rpm for 10 min, and the collected solid was calcined at 400 °C for 4 h to yield the final MgO NPs [14, 32].

Second, as for AgHap NPs, they were prepared by Nano gate, Cairo as the following, the (PO_4_)_6_(OH)_2_, ceramic powder, was prepared (Ca/P molar ratio: 1.67) using calcium nitrate [Ca (NO_3_)_2_•_4_H_2_O] and diammonium hydrogen phosphate [(NH_4_)_2_HPO_4_] by coprecipitation. For silver doped hydroxyapatite nanoparticles, the ratio [Ca + Ag]/P was 1.67. Silver nitrate AgNO_3_ and calcium nitrate[Ca (NO_3_)_2_•_4_H_2_O] were dissolved in deionized water to a final volume of 300mL [Ca + Ag]-containing solution. Diammonium hydrogen phosphate [(NH_4_)_2_HPO_4_] was dissolved in deionized water to a final volume of 300 mL P-containing solution. The [Ca + Ag]-containing solution was stirred at 100 °C for 30 min. The P-containing solution with a pH of 10 (adjusted pH NH_3_) added drop by drop into the [Ca + Ag]-containing solution and stirred for 2 h. The pH value was constantly adjusted at 10 during the reaction. Afterwards, the precipitate was washed several times with deionized water. The resulting material was dried at 100 °C for 72 h [33].

While, ChHap NPs were prepared by Nano gate, Cairo as following, the narrowly dispersed spherical hydroxyapatite (Hap)NPswere prepared through the dropwise addition of [(NH_4_)_2_HPO_4_] to [Ca (NO_3_)_2_•_4_H_2_O] in an alkaline pH under vigorous agitation. Then the Hap NPs were coated with chitosan by the gradual addition of a chitosan solution under ultrasonication. The resulting precipitate was aged, washed, centrifuged, and then dried in a vacuum oven at 60 °C [34].

Finally, ZnO NPs were prepared by Laser Institute, Cairo university, as following wet chemical method using zinc nitrate and sodium hydroxide as precursors and soluble starch as stabilizing agent was used for ZnO NPs preparation [35].

### Characterization of nanoparticles

The preparedNPs were characterized for size and stability using JEOL JEM-2100 high resolution TEM and zeta potential by photon correlation spectroscopy using particle size analyzer dynamic light scattering (DLS) (Zetasizer Nano ZN, Malvern Panalytical Ltd, United Kingdom), in addition to UV-Vis spectrophotometry (T80^+^ PG instrument). Samples were analyzed in triplicate [36].

### Bacterial standard strains and isolates

Standard strains of *E. coli* (ATCC 25922) and *K. pneumoniae*(ATCC 13883) were purchased from the Egyptian microbial culture collection (EMCC), Faculty of agriculture, Ain Shams University.

A total of 120 clinical isolates were collected from the bacteriology laboratory in Qasr El-Eni hospital in Cairo, Egypt. Luria-Bertani media is used for bacterial growth in all experiments, pH was maintained near neutral at 6.8-7, unless mentioned elsewhere.

### Identification of clinical isolates

The 120 clinical isolates were identified by Gram staining, microscopicexamination, and standard biochemical testing methods [37]. Sample sources are mentioned in detail in Table S1.

Antibiotic susceptibility ofcollected clinical isolates was determined using the standard agar disc diffusion susceptibility testing technique using antibiotic discs mentioned in Tables S2 following the standard procedures of CLSI 2022 [38].

### Identification of carbapenem resistance pattern

First the carbapenemase-producing isolates were detected using modified carbapenem inactivation method (mCIM) [39]. Briefly, the procedure was carried outfollowing the standard procedures of CLSI [40]. For each isolate to be tested, a 1-µL loopful of bacteria from an overnight blood agar plate was emulsified in 2 mL tryptic soya broth (TSB), followed by addition of 10-µg meropenem disk to each tube and incubation at 35 °C ± 2 °C in ambient air for 4 h ± 15 min. Just before or immediately following completion of the TSB-meropenem disk suspension incubation, a Mueller Hinton agar (MHA) plate was inoculated with *E. coli* ATCC (25922) as for the routine disk diffusion procedure. Finally, the meropenem disk was removed and placed on the MHA plate and incubated at 35 °C ± 2 °C in ambient air for 18–24 h [39].

Second, the phenotypic detection of carbapenemasetype was carried out using EDTA modified carbapenem inactivation method (eCIM) [41].The procedures followed the mCIM protocol, with the only modification being the addition of 20 µLof 0.5 M EDTAto the 2 mL TSB tube to obtain a final concentration of 5 mM EDTA.

### Assessment of antibacterial activity of tested NPs

The minimum inhibitory concentrations (MICs) of the tested NPswere determined using the standard microbroth two-fold serial microdilution technique in microtiter platesasdescribed before [42, 43] with minormodifications. Firstly, NPs dispersion conditions have been optimized as previously reported [44]. Briefly, overnight cultures of the tested bacterial isolates were adjusted to a final concentration of 1.5 × 10^5^ CFU.ml^−1^, and the starting concentrationsof NPs were 8, 10, 10 and 20 mg/mL for MgO NPs, AgHap NPs, ZnO NPs, ChHap NPs, respectively.Afterwards, the microtiter plates were incubated at 37 °C for 18–20 h. All experiments wererepeatedin triplicates.

### Assessment of antibacterial activity of vitamin D combination with NPs

Determination of MIC for Vitamin D was performed against one *E. coli* isolate using microdilution technique as described above. The effect of the combination of the promising NPs (AgHap NPs and MgO NPs)with Vitamin D was evaluated by checker-board assay. We usedvarying concentrations of Vitamin D and NPs across the x and y axis of 96 well plates, and the fractional inhibitory concentration (FIC) index was determined [45, 46]. Experiments were done in triplicates.

### Evaluating the antibacterial mechanism of action of the NPs

#### Effect of the Nps on cell wall integrity

Samples were prepared according to Hamedet al. [47] with some modifications. Bacterial suspension for one *E. coli*isolate was treated with the most promising NPs,1 mg. mL^−1^of MgO NPs or 1.35 mg. mL^−1^ of AgHap NPs, then incubated for 6 and 12 h. After that, samples were centrifuged for 20 min at 4000 rpm at 18 °C, and the supernatant was discarded and subsequently, 1mLglutaraldehyde was added and left for 4 h. Finally, the samples were re-centrifuged for 20 min at 4000 rpm at 18 °C then 1 mL of PBS was added, andthe interaction between NPs and microbial cells was examined by TEM. A negative control of the untreated bacterial cells was also prepared in the same manners and examined.

#### Molecular docking study

The molecular docking study was fulfilled by PYRX 0.8 that applies both AutoDock 4 and AutoDock Vina for best validation of the docking process. The crystal structure of *E. coli* DNA gyrase was downloaded from the protein data bank; 3G7E in complex with an inhibitor [48]. All tested NPs were built andminimized and protein were prepared. The free energy of binding (ΔG) and the binding affinity were computed (Kcal/mol) during the docking process. The root of mean square fluctuation (RMSF)was computed over 50 nanoseconds(ns) period of molecular dynamics(MD) simulation time.

### Assessment of cytotoxicity profiles of Nps, vitamin D and their combination

The MTT (3-[4,5-dimethylthiazole-2-yl]−2,5-diphenyltetrazolium bromide) cell viability assay was performed usingthe human hepatocellular carcinoma cell line (HepG2)to evaluate the cytotoxic effects of the most promisingNPs, Vitamin D, and their combination. The experiment was conducted at VACSERA Co., Egypt. In brief, 5 × 10⁴ cells per well were seeded in a serum-free medium in a 96-well flat-bottom microplate and treated with 20 µL of various concentrations of the compounds for 48 h at 37 °C in a 5% CO_2_ incubator. After a 4hrincubation period, the medium was removed, and 40 µL of MTT solution was added to each well [49, 50]. Absorbance was measured at 570 nm using a microplate ELISA reader. The experiment was performed in triplicate.

### Statistical analysis

The Means and standard deviations from both biological and technical replicates were calculated using Origin Prolab 2022, USA.

## Results

### Characterization of nanoparticles

**Fig. 1:**
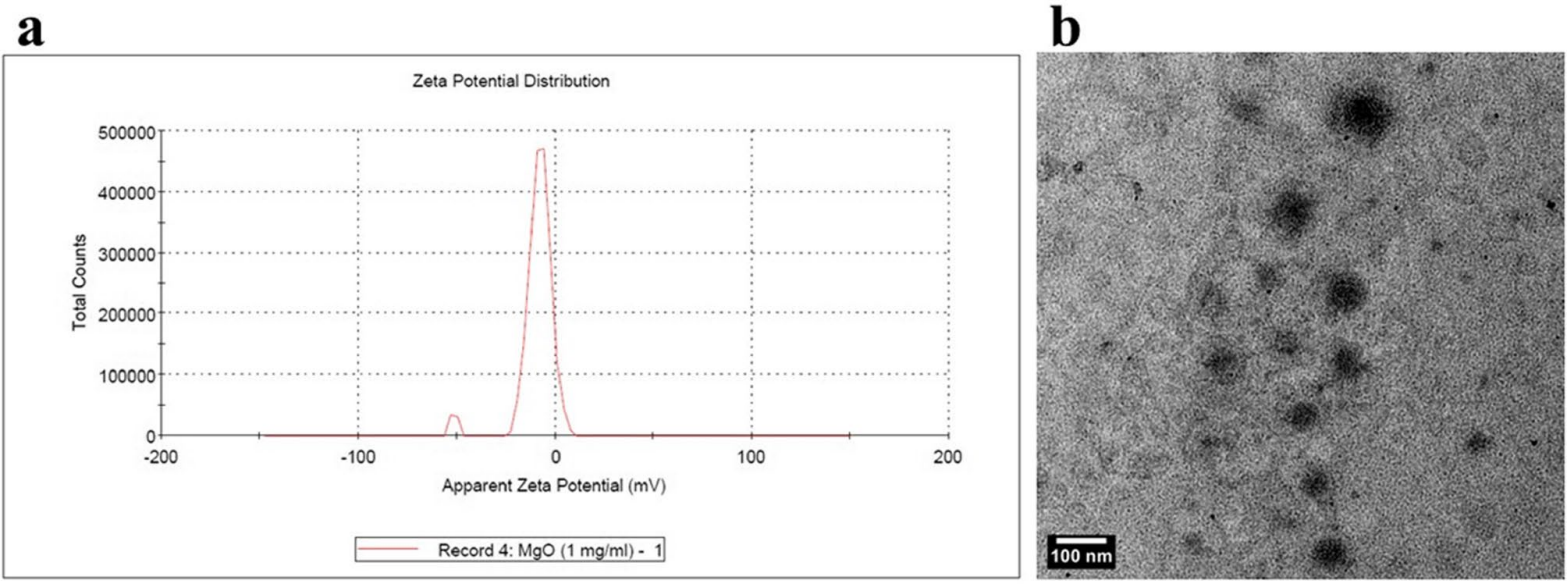
Stability, size and shape characterization for MgO NPs. **a**: Zeta potential graph for MgO NPs, **b**: TEM image of MgO NPs showing spherical particles with an average size of 10 nm

The stability of the nanoparticles (NPs) was evaluated by measuring the zeta potential or UV spectroscopy. Transmission electron microscope(TEM) was used to obtain detailed images of the NPs to characterize morphology and size distribution. The zeta potential of magnesium oxide NPs (MgO NPs) was found to be −4.59 ± 0.66 mV. The TEM images revealed that the MgO NPs were spherical in shape with an average size of 10 nm, as shown in (Fig. 1).

**Fig. 2:**
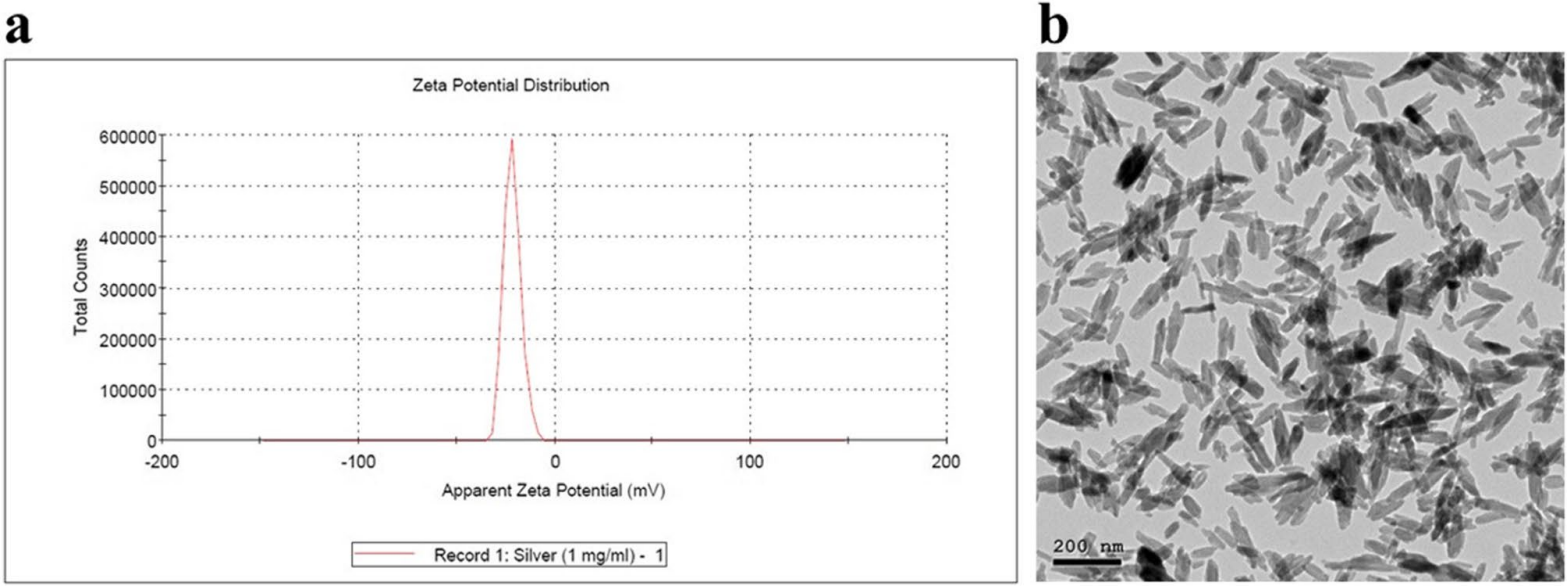
Stability, size and shape characterization for AgHap NPs. **a**: Zeta potential graph for AgHap NPs, **b**: TEM image for AgHap NPs showing needle shaped particles with an average size of 133.3 nm

For silver hydroxyapatite NPs (AgHap NPs), the zeta potential was − 22.1 ± 0.81 mV. The TEM images of the AgHap NPs revealed that the NPs had a needle-like structure, with an average size of 133.3 nm, as shown in (Fig. 2).

**Fig. 3:**
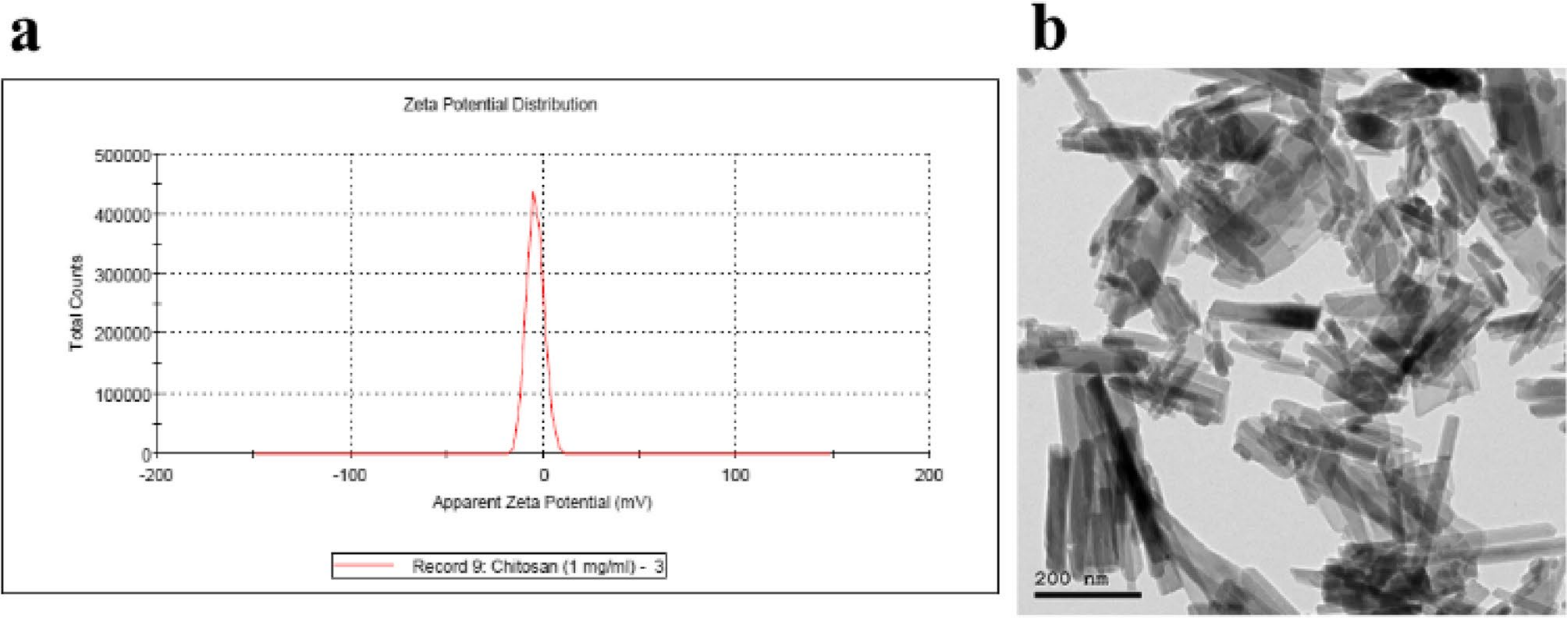
Stability, size and shape characterization for ChHap NPs. **a**: Zeta potential graph for ChHap NPs, b: TEM image for ChHap NPs showing needle shaped particles with an average size of 120 nm

For chitosan hydroxyapatite NPs(ChHap NPs), the zeta potential was − 10.4 ± 0.89 mV. The TEM images of the ChHap NPs revealed that the NPs had a needle-like structure, withan average size of 120 nm as shown in (Fig. 3).

**Fig. 4:**
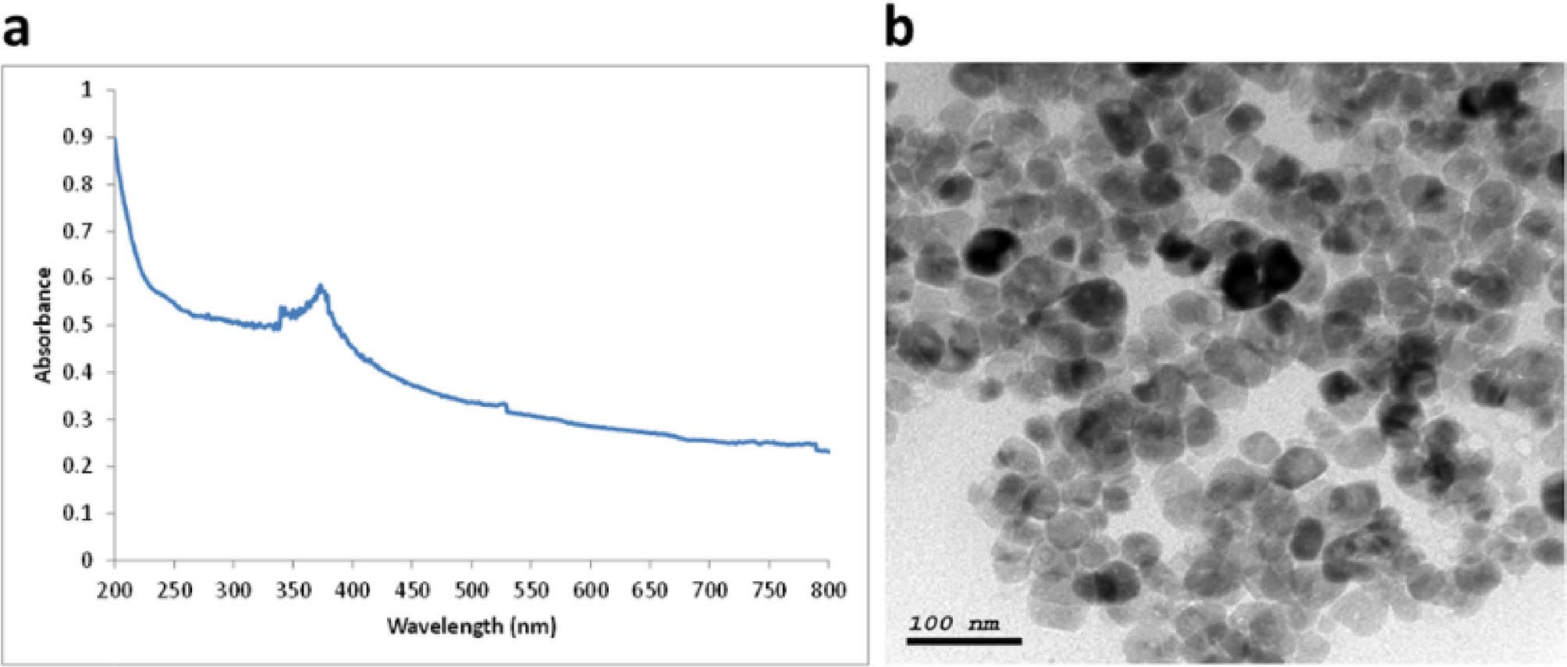
Stability, size and shape characterization for ZnO NPs. **a**: UV absorbance spectrum for ZnO NPs, **b**: TEM image for ZnO NPs showing spherical shaped particles with an average size of 50 nm

For zinc oxide NPs (ZnO NPs), UV-absorption spectra peak was at 370 nm as shown in **(**Fig. 4**)**. The TEM images of the ZnO NPs revealed that the NPs were spherical in shape, with an average size of 50 nm as shown in **(**Fig. 4**)**.

### Identification of clinical isolates

Out of the collected 120 clinical isolates, 118 were used in this study were identified as carbapenem-resistant Gram-negative rods, distributed as: 59 isolates *K. pneumoniae*, 36 isolates *A. baumannii*, 5 isolates *P. aeruginosa*,2 isolates *E. aerogenes*, one isolate *P. mirabilis*, one isolate* Citrobacter* species, and 14 isolates* E.coli* as shown in Tables S3,while the other 2 carbapenem-sensitive isolates were excluded from the study.

Carbapenem-resistant isolates included 45 isolates non-carbapenemase producers and 73 ones carbapenemase producers (CP). The types of carbapenemases were identified using the EDTA inactivation method, which revealed that 65 isolates were metallo-β-lactamases while 8 isolates were serine carbapenemases.

### Antibacterial activityof NPs and vitamin D and their combination

The minimum inhibitory concentration (MIC) value of ZnO NPs was 937.5 µg/mL against one *E. coli* isolate, and for AgHap NPs against all the bacterial isolates was 1.875 mg. mL^−1^, while MgO NPs had MIC valuesof 1.5, 1.5, 1.5, 1.5, 1.5, 0.75, and 0.375 mg. mL^−1^ against *E. coli*, *K. pneumoniae*, *E. aerogenes*,* Citrobacter *species, *P. mirabilis*, *P. aeruginosa* and *A. baumannii*, respectively, and finally ChHap NPs had MIC value of 234.4 µg/mL against one *E. coli* isolate. On the other hand, the MIC value of Vitamin D alone was 0.234 mg. mL^−1^ against one *E. coli *isolate.

The effect of the combination of the promising NPs (AgHap NPs and MgO NPs) with Vitamin D was determined using a checker-board assay. The fractional inhibitory concentration (FIC) index for MgO NPs and Vitamin D was 0.75, while for AgHap NPs and Vitamin D it was 0.52.

FICI= (MIC of drug A in combination/MIC of drug A alone) + (MIC of drug B in combination/MIC of drug B alone).

FICI of MgO NP= (0.75 mg. mL^−1^/1.5 mg. mL^−1^) + (0.0585 mg. mL^−1^/0.234 mg. mL^−1^).

FICI of AgHap NP= (0.9375 mg. mL^−1^/1.875 mg. mL^−1^) + (0.0037 mg. mL^−1^/0.234 mg. mL^−1^).

### The antibacterial mechanism of action of the NPs: effect of the NPs on cell wall integrity

**Fig. 5:**
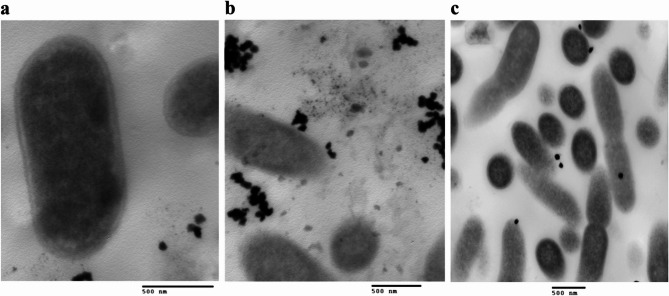
Images for *E. coli *isolate before and after treatment with MgO NPs as revealed by TEM.a: represents the control, b and c: represent*E. coli*treated with MgO NPs after 6 and 12 h, respectively

**Fig. 6:**
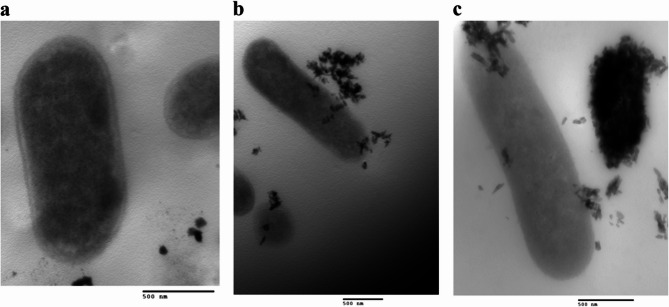
Images for *E. coli *isolate before and after treatment with AgHap NPs as revealed by TEM.a: represents the control, b and c: represent *E. coli*treatedwith AgHap NPsafter 6 and 12 h, respectively

A comparison and analysis of TEM images of the control and treated *E. coli *isolate after 6 and 12 h of NPs treatment showed that the promising NPs (AgHap NPs and MgO NPs) disrupted the cell wall integrity. After 6 h, the NPsadhered to the cell wall, entered the cell, and accumulated within it. By 12 h, cell deformation was observed, including elongation and appearance of ghost cells. Ultimately, the cells ruptured, and their contents were released, as shown in the images(Figs. 5 and 6).

### The cytotoxicity profiles of nps, vitamin D and their combination

**Fig. 7:**
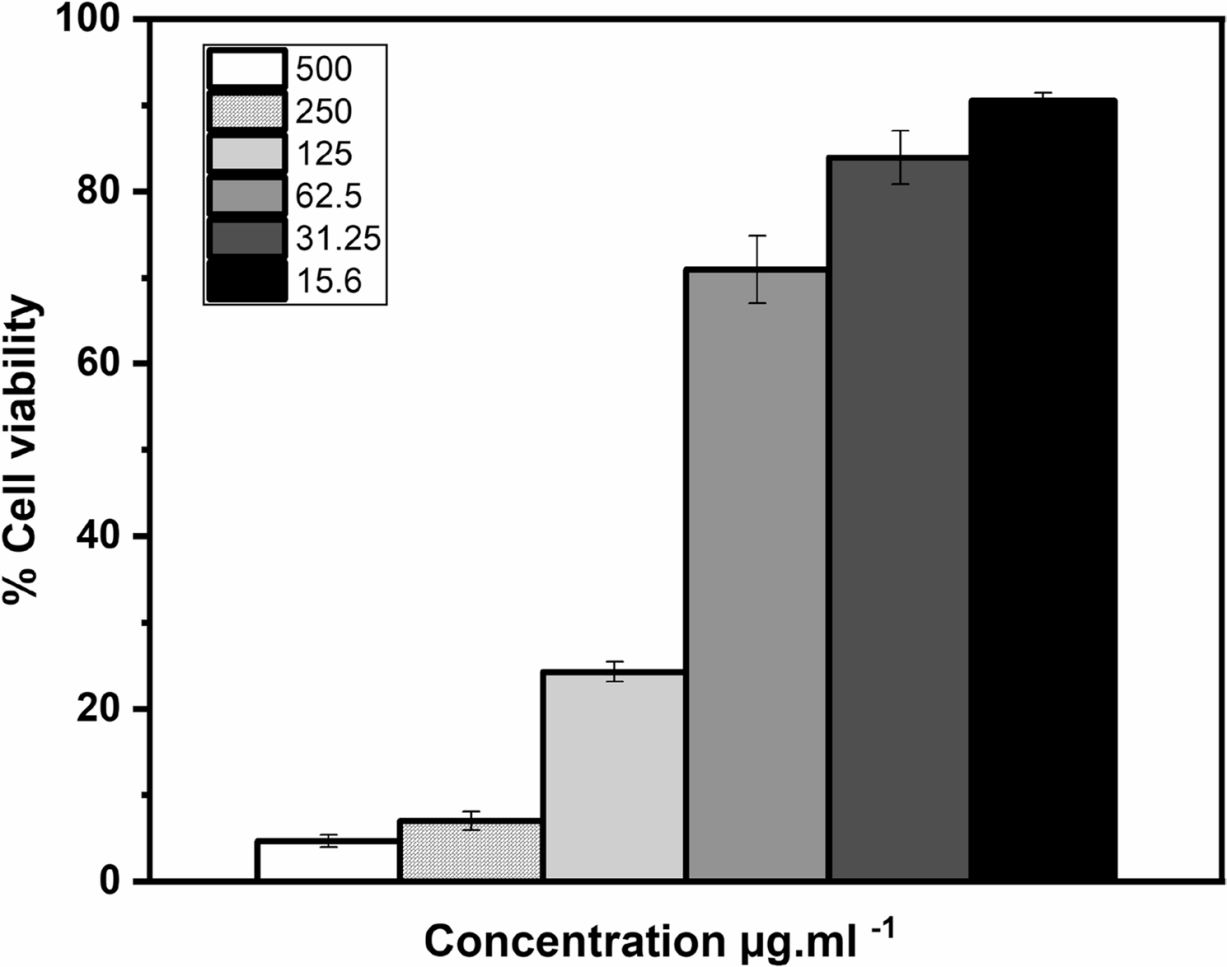
Cytotoxicity profiles of Vitamin D against HepG2 cells

**Fig. 8:**
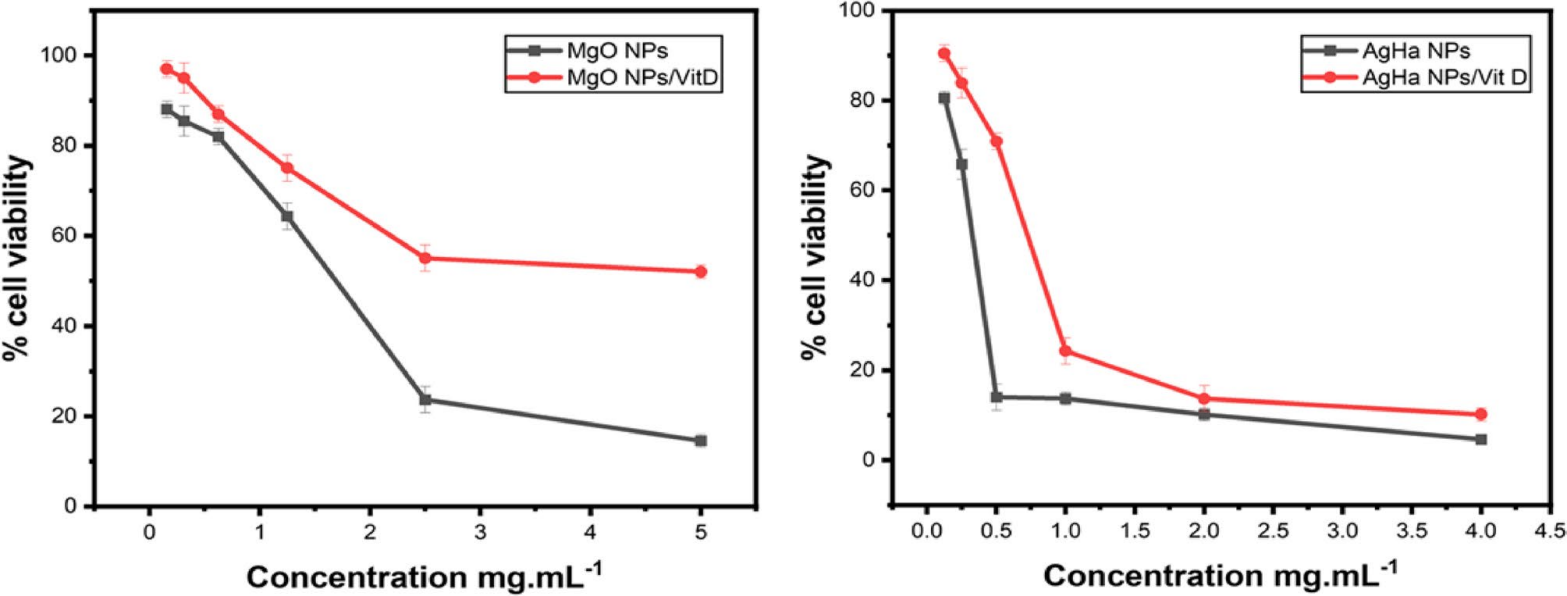
Cytotoxicity profilesof MgO NPs and AgHap NPs and each one combinedwith Vitamin D against HepG2 cells

The impact of the promising NPs (AgHap NPs and MgO NPs), Vitamin D, and their combinations on the proliferation of human hepatocellular carcinoma cell line (HepG2) was examined after 48 h of incubation using the MTT assay. Vitamin D alone resulted in about 7% cell viability near its MIC and the cell viability increased as Vitamin D concentrations decreased (Fig. 7). While the results shown in (Fig. 8**)**, indicated that the NPs maintained approximately 40–85% cell viability at their MIC values. Additionally, combining Vitamin D, at a fixed concentration of 40 µg. mL^−1^, with the tested NPs improved the cell viability compared to the individual treatments.

### Molecular docking study

**Fig. 9:**
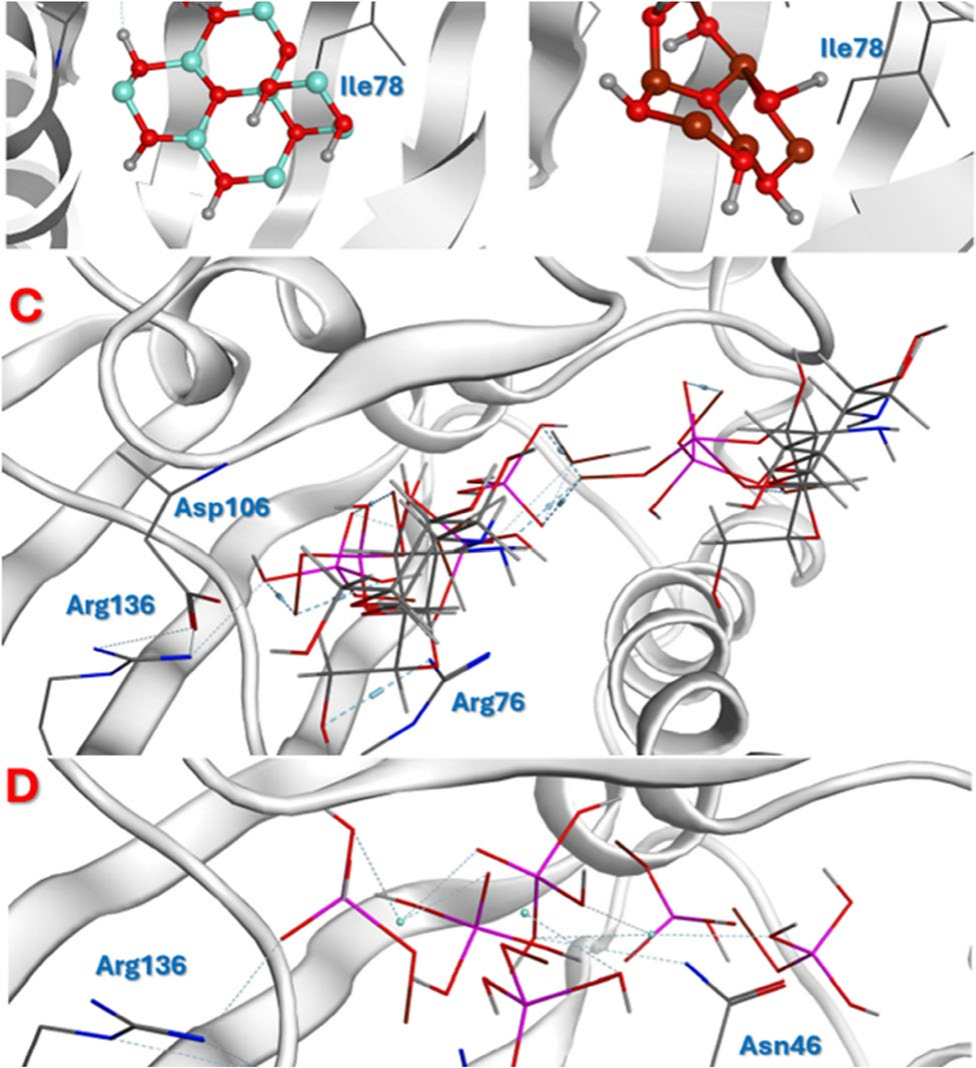
The best docking pose of **A**) ZnO NPs, **B**) MgO NPs, ChHap NPs, **D**) AgHap NPs against DNA-gyrase of *E. coli*

The tested NPs were docked and their interaction profile was analyzed. ZnO NPs was built as a hexagonal structure as previously reported [51] and upon docking it showed two hydrogen bonds with Asp73 and Thr165 (Fig. 9A). Where MgO NPs showed only one hydrogen bond between Mg and Asp73 (Fig. 9B). The negative charges of the phosphate groups found in both ChHap NPs, and AgHap NPs enabled them to form ionic bonds with two positively charged residues; Arg136, Arg76 (Fig. 9C and D) respectively. The free energy of binding values for ChHap NPs, and AgHap NPs were almost the same (−9.61, −9.58 kcal/mol) respectively. The validation of docking was performed by computing the binding affinity that showed the same ranking (Table 1).

**Table 1:** Docking results of the tested NPs against *E. coli* DNA gyrase

	Free energy of binding ΔG Kcal/mol	Binding affinity	Interacted residues
Zinc oxide	−8.35	12.18	Asp73 and Thr165
Magnesium oxide	−10.75	15.97	Asp73
Chitosan hydroxyapatite	−9.61	13.47	Arg136, Arg76
Silver hydroxyapatite	−9.58	13.38	Arg136, Arg76

**Fig. 10:**
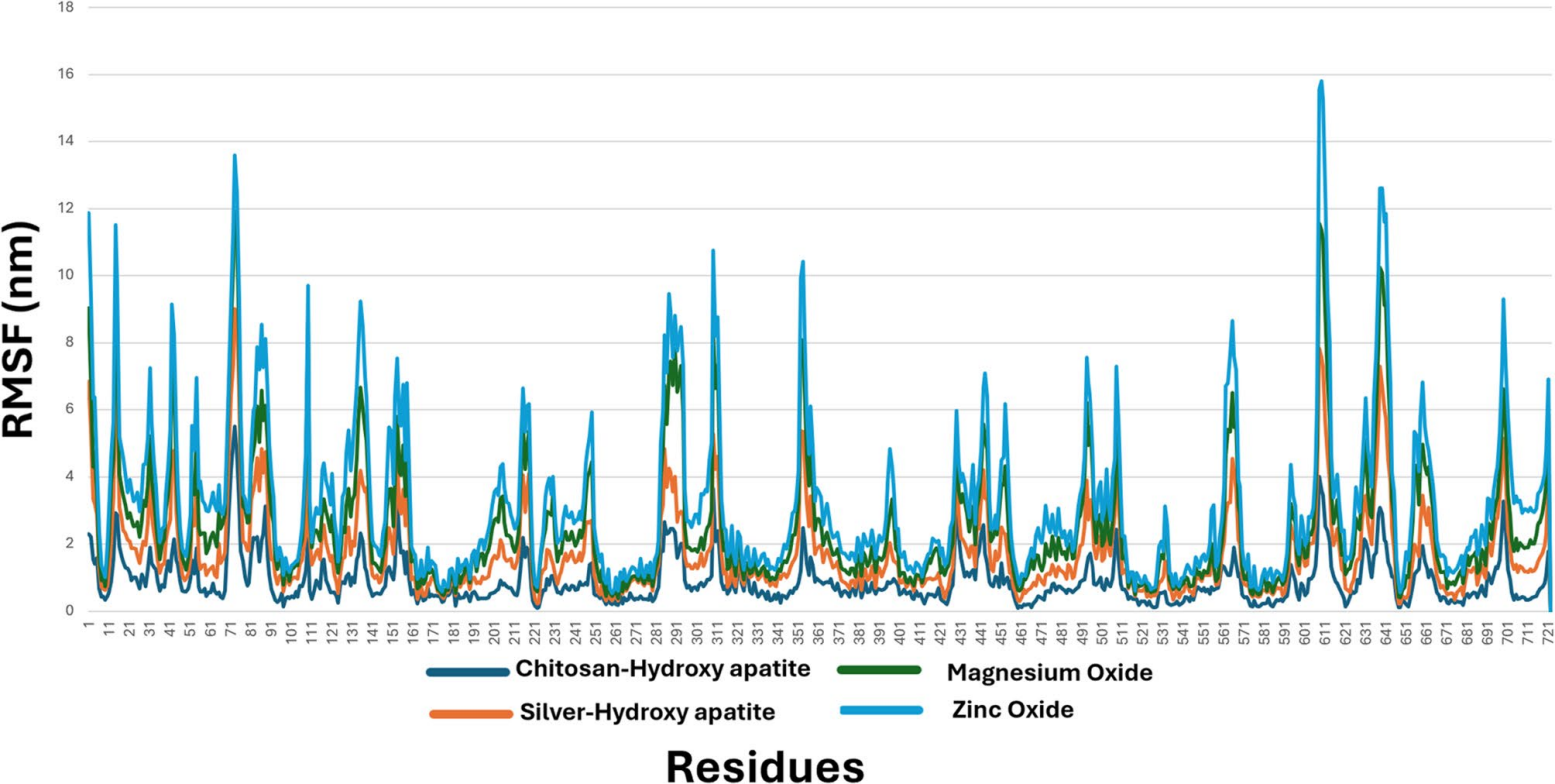
RMSF of (**A**) ChHap NPs (Dark Blue). (**B**) AgHap NPs (Orange). (**C**) MgO NPs (Green). (**D**) ZnO NPs (Blue)

The best pose of each of the docked NPs was kept into the active site.The complexes were subjected to dynamic simulations over 50nanoseconds(ns). Theflexibility of amino acid residues and the distance between the atoms were computed using root of mean square fluctuation (RMSF). Complex showed the lowest deviation was ChHap NPs (Fig. 10, dark blue line), however ZnO NPs showed the highest deviation when compared to the other compounds **(**Fig. 10**)**. The binding stability of the tested NPs over the 50 ns period was confirmed by RMSF. Furthermore, ChHap NPs (Dark Blue line) and AgHap NPs (Orange line) were very close to each other with low deviation which reflects the strength of their ionic interactions within the active site as discussed in the docked results.

## Discussion

At the time being, antimicrobial resistance (AMR) is a worldwide substantial public health crisis; thereby a prompt solution should be developed [1, 2]. Among resistant pathogens, carbapenem-resistant Gram-negative bacteria(CRGNB)—such as *K. pneumoniae*, *A. baumannii*, and *P. aeruginosa*—are of greater clinical concern compared to their Gram-positive counterparts [52]. According to World Health Organization (WHO), there are 13 resistant bacteria that pose a high risk to human health, the first and second ranks include carbapenem-resistant *A. baumannii*,* P. aeruginosa* and Enterobacteriaceae [53].

Nanotechnology offers a promising approach to combat this growing threat, when reduced to the nanoscale, metals and metal oxide nanoparticles (NPs) demonstrate enhanced characteristics, including high reactivity, improved mechanical properties, and unique optical behaviors [54]. These properties are leveraged in various industries, from medical treatments to advanced energy systems [13, 17].

Unlike previous studies that often focus on a single nanoparticle type or rely solely on *in -vitro* assays, this work combines predictive in silico modeling with biological validation, offering a more mechanistic and comprehensive understanding of nanoparticle–bacteria interactions. Moreover, the inclusion of both standard bacterial strains and clinically isolated multidrug-resistant (MDR) pathogens enhances the clinical relevance of the findings. This integrated strategy not only broadens the knowledge of nanoparticle-based antimicrobials but also supports the rational design of novel nanoformulations targeting drug-resistant infections.

Several ways exist for nanoparticle synthesis, including traditional chemical methods and biological (“green”) approaches that employ microbial reductase enzymes or plant-derived reducing agents like flavonoids and phenols [43, 55]. However, green synthesis still faces significant challenges in achieving precise control over nanoparticle size, shape, and purification. Consequently, chemical reduction remains the most straightforward technique for tailoring nanoparticle morphology and size distribution.

The size of NPs plays a crucial role in determining their physicochemical properties, including surface area, reactivity, and stability. In this study, the size and stability of magnesium oxide (MgO NPs), zinc oxide (ZnO NPs), chitosan hydroxyapatite (ChHap NPs) and silver hydroxyapatite (AgHap NPs) were determined using zeta sizer or UV spectroscopy and transmission electron microscope(TEM) imaging, which are reliable methods for assessing particle size, distribution, and surface charge. We prepared the MgO NPs, ZnO NPs, ChHap NPs and AgHap NPs at sizes of 10 nm, 50 nm, 120 nm and 133 nm, respectively. The nanoscale particle sizes achievedare considered almost optimal because as the size of NPs decreases, the surface-to-volume ratio increases significantly, which enhances their ability to interact with microorganisms. Smaller NPs possess a larger surface area, allowing for greater contact with microbial cells, which often leads to improved antimicrobial activity. This heightened surface interaction is crucial for the effectiveness of NPs in various applications, including bacterial control. However, controlling particle size and distribution is challenging, as smaller particles are more susceptible to oxidation, which can impact their stability and functionality [56]. Furthermore, controlling the stability of NPs under different conditions remains essential for maintaining their effectiveness, as their reactivity is highly influenced by both size and surface chemistry [57, 58].

We focused on CRGNB that cause a plethora of severe infections, including soft tissue infections, pneumonia, endocarditis, complicated urinary tract infections, respiratory infections, and complicated intra-abdominal infections [59]. This is largely due to the complex resistance mechanisms employed by Gram-negative organisms, including the production of carbapenemases, efflux pumps, porin mutations, and the horizontal transfer of resistance genes through plasmids [60]. These bacteria are often associated with high morbidity and mortality, particularly in intensive care units, and have shown limited susceptibility to remaining treatment options. A study conducted in Egypt reported that 20% of Gram-negative isolates demonstrated carbapenem resistance, with *K. pneumoniae *being the most resistant species [5].

In contrast, carbapenem resistance in Gram-positive bacteria- such as *E. faecium *or *S. aureus* is relatively uncommon and generally less threatening in scope and spread [61]. The intrinsic resistance mechanisms in Gram-positive bacteria differ significantly and often do not involve carbapenemases, making them somewhat less adaptable in spreading carbapenem resistance compared to Gram-negatives [62].​

Most of these strains are still susceptible to polymyxins and tigecycline, but these antibiotics do not have the desired pharmacokinetic properties. As a result, there is still a high mortality rate [63, 64].

Initially, more than 120 clinical isolates were collected, in addition to standard strains of *E. coli* (ATCC 25922) and *K. pneumoniae *(ATCC 13883). Minimum inhibitory concentration (MIC) values of tested NPs were determined by two-folds serial dilution technique. The results showed that AgHap NPs exhibited a uniform MIC value of 1.875 mg/mL across all tested bacterial isolates. In contrast, MgO NPs displayed varying MIC values depending on the bacterial strain: 1.5 mg. mL^−1^ for *E. coli*, 1.5 mg. mL^−1^ for *K. pneumoniae*, and 0.375 mg. mL^−1^ for *A. baumannii*. These findings indicate that MgO NPs demonstrated stronger antimicrobial activity against *A. baumannii*, a common carbapenem-resistant pathogen, compared to *E. coli* and *K. pneumoniae*, which are also important clinical pathogens [53, 65]. The differences in MIC values suggest that particle composition and bacterial resistance mechanisms significantly influence the effectiveness of nanoparticles, underscoring the importance of strain-specific antimicrobial evaluations. On the other hand, ZnO NPs and ChHap NPs had the least antibacterial activity, as they inhibited the growth of only one bacterial isolate. This may be related to the NPs size and shape; as they affect antimicrobial activity, for example spherical metal NPs has high antibacterial property compared to other shapes depending on ion release [66].

The cytotoxicity of the most promising NPs (MgO NPs and AgHap NPs) was assessed near the MIC values using the MTT assay with human hepatocellular carcinoma cell line (HepG2), and preliminary results indicated that cell viability ranged from 40 to 85%. These findings align with previous studies suggesting that the toxicity of NPs can vary depending on concentration and exposure time [67]. Based on prior experience, it was anticipated that the inclusion of an adjunct antioxidant such as Vitamin D or N-acetyl cysteine could enhance cell viability by reducing oxidative stress [30, 31, 68]. In this study, Vitamin D exhibited partial synergism with the MgO NPs and AgHap NPs, effectively reducing cytotoxicity when compared to the NPs alone, as seen in similar research [69]. Interestingly, the reduction in cytotoxicity was more pronounced when Vitamin D was combined with MgO NPs than with AgHap NPs. This suggests that the interaction between Vitamin D and certain NPs may be influenced by the physical properties of the NPs, such as size and surface charge, which can affect their bioactivity and cellular uptake [70].

The effect of the MgONPs and AgHap NPs on bacterial cell wall integrity was also evident in TEM images. Upon treatment, NPs attached to the bacterial cell wall, entered the cell, and accumulated inside, causing the disruption of cell wall integrity. Over time, this led to the leakage of cellular contents, resulting in cell death. This mechanism of action supports the growing evidence that NPs, particularly those with antimicrobial properties, can act as effective alternatives to traditional antibiotics [71–73].

The reduced potential for resistance development in bacteria when exposed to NPs further underscores their promise as a viable option for combating antibiotic-resistant pathogens [74].

By bridging computational predictions with biological evaluation, this study offers a comprehensive model for screening and optimizing nanomaterials in combating MDR pathogens.The docking study was performed to predict the binding affinity and strength of the tested NPs against bacterial *E. coli *DNA gyrase and to correlate the results to the antibacterial activity. This target was selected as it was reported that MgO NPs, ZnO NPs, ChHap NPs, and AgHap NPs have direct inhibitory activity of this enzyme that is proportional to its antibacterial action [75–78]. The top ranked free energy of binding (ΔG) was achieved by MgO NPs which can be correlated with its antibacterial activity. These outcomes contribute meaningfully to the scientific community by advancing nanoparticle-based antimicrobial delivery systems and support their potential translation into clinical settings. Ultimately, such approaches could enhance therapeutic efficacy, reduce reliance on conventional antibiotics, and yield significant positive implications for human health and the global fight against antimicrobial resistance.

## Conclusion

Taken together, magnesium oxide (MgO NPs)and silver hydroxyapatite (AgHap NPs) nanoparticlesshowed strong antibacterial effects against carbapenemresistant Gramnegative isolates, with acceptable cytotoxicity near minimum inhibitory concentration (MIC) levels, particularly when used in combination with adjunctive agents like Vitamin D. Molecular docking and TEM analyses suggest their mechanism involves enzyme inhibition and disruption of bacterial cell wall integrity. These results support their potential as alternative therapeutic agents, warranting further *in-vivo* evaluation.

### Future work

While this study provides valuable insights into the antimicrobial potential of various nanoparticles against carbapenem-resistant Gram-negative bacteria, further research is recommended to expand the scope and depth of understanding. Future studies should explore the molecular mechanisms of nanoparticle-bacteria interactions using advanced in-silico techniques such as molecular dynamics simulations and quantitative structure–activity relationship (QSAR) models. Additionally, evaluating the cytotoxicity and biocompatibility of these nanoparticles in mammalian cell lines is essential to assess their clinical safety. In-vivo studies and the development of nanoparticle-based delivery systems or coatings for medical devices could further enhance their practical applications. Combining nanotechnology with synergistic agents such as antibiotics or peptides may also offer promising avenues for overcoming bacterial resistance.

## Supplementary Information

Supplementary Material 1

## Data Availability

All the datasets generated or analyzed during this study are included in this manuscript and its supplementary information files.

## Abbreviations

ΔG: Free energy of binding
AgHap: Silver hydroxyapatite
AMR: Antimicrobial resistance
ChHap: Chitosan hydroxyapatite
CLSI: Clinical and laboratory standard institute
CP: Carbapenemase producers
CRE: Carbapenem-resistant Enterobacteriaceae
CRGNB: Carbapenem-resistant Gram-negative bacterial strains
DLS: Dynamic light scattering
eCIM: EDTA modified carbapenem inactivation method
FDA: Food and Drug Administration organization
FIC: Fractional inhibitory concentration
H: Hour
mCIM: Modified carbapenem inactivation method
MD: Molecular dynamics
MDR: Multidrug resistant
MgO: Magnesium oxide
MHA: Muller Hinton agar
MIC: Minimum inhibatory concentration
MIN: Minute
NP: Nanoparticle
Ns: Nanoseconds
QSAR: Quantitative structure–activity relationship models
ROS: Reactive oxygen species
RMSF: Root of mean square fluctuation
RPM: Revolutions Per Minute
TEM: Transmission electron microscope
TSB: Tryptic soya broth
WHO: World Health Organization
ZnO: Zinc oxide
