# Supplementary material for: Assessment of MgO, ZnO, chitosan hydroxyapatite and silver hydroxyapatite nanoparticles against carbapenem-resistant Gram-negative Egyptian clinical isolates: a combined in-silico and in-vitro study
Source: BMC Microbiol. 2025 Oct 6;25:632. doi: 10.1186/s12866-025-04364-y (PMC12502137; doi:10.1186/s12866-025-04364-y)
Supplement: Supplementary file 1 — Supplementary Material 1 [file 12866_2025_4364_MOESM1_ESM.docx]

Supplementary documents:

**Table S1: Distribution of isolates, species and sample source.**

| Isolate No. | Micro organism | Source |
| --- | --- | --- |
| 1 | *Klebsiella pneumoniae* | Urine |
| 2 | *Pseudomonas aeruginosa* | Blood Culture |
| 3 | *Klebsiella pneumoniae* | Pus |
| 4 | *Klebsiella pneumoniae* | Chest Culture |
| 5 | *Klebsiella pneumoniae* | Blood Culture |
| 6 | *Pseudomonas aeruginosa* | Urine |
| 7 | *Klebsiella pneumoniae* | Wound |
| 8 | *Klebsiella pneumoniae* | Endo Culture |
| 9 | *Pseudomonas aeruginosa* | Wound |
| 10 | *Klebsiella pneumoniae* | Wound |
| 11 | *Klebsiella pneumoniae* | Endo Culture |
| 12 | *Klebsiella pneumoniae* | Wound |
| 13 | *Klebsiella pneumoniae* | Urine |
| 14 | *Pseudomonas aeruginosa* | Urine |
| 15 | *Klebsiella pneumoniae* | Blood Culture |
| 16 | *Klebsiella pneumoniae* | Wound |
| 17 | *Klebsiella pneumoniae* | Blood Culture |
| 18 | *Klebsiella pneumoniae* | Blood Culture |
| 19 | *Klebsiella pneumoniae* | Blood Culture |
| 20 | *Klebsiella pneumoniae* | Blood Culture |
| 21 | *Klebsiella pneumoniae* | Pus |
| 22 | *Escherichia coli* | Pus |
| 23 | *Klebsiella pneumoniae* | Blood Culture |
| 24 | *Klebsiella pneumoniae* | Pus |
| 25 | *Escherichia coli* | Wound |
| 26 | *Escherichia coli* | Ear Discharge |
| 27 | *Citrobacter* sp. | Pus |
| 28 | *Escherichia coli* | Urine |
| 29 | *Klebsiella pneumoniae* | Blood Culture |
| 30 | *Klebsiella pneumoniae* | Pus |
| 31 | *Klebsiella pneumoniae* | Pus |
| 32 | *Klebsiella pneumoniae* | Endo Culture |
| 33 | *Klebsiella pneumoniae* | Blood Culture |
| 34 | *Klebsiella pneumoniae* | Wound |
| 35 | *Klebsiella pneumoniae* | Urine |
| 36 | *Acinetobacter baumannii* | Sputum |
| 37 | *Acinetobacter baumannii* | Blood Culture |
| 38 | *Klebsiella pneumoniae* | Pus |
| 39 | *Klebsiella pneumoniae* | Pus |
| 40 | *Escherichia coli* | Urine |
| 41 | *Pseudomonas aeruginosa* | Urine |
| 42 | *Klebsiella pneumoniae* | Urine |
| 43 | *Klebsiella pneumoniae* | Pus |
| 44 | *Klebsiella pneumoniae* | Blood Culture |
| 45 | *Escherichia coli* | Urine |
| 46 | *Acinetobacter baumannii* | Urine |
| 47 | *Acinetobacter baumannii* | Sputum |
| 48 | *Acinetobacter baumannii* | Blood Culture |
| 49 | *Acinetobacter baumannii* | Blood Culture |
| 50 | *Acinetobacter baumannii* | Blood Culture |
| 51 | *Acinetobacter baumannii* | Tissue |
| 52 | *Acinetobacter baumannii* | Tissue |
| 53 | *Proteus mirabilis* | Blood Culture |
| 54 | *Acinetobacter baumannii* | Sputum |
| 55 | *Acinetobacter baumannii* | Sputum |
| 56 | *Escherichia coli* | Pus |
| 57 | *Acinetobacter baumannii* | Blood Culture |
| 58 | *Acinetobacter baumannii* | Blood Culture |
| 59 | *Acinetobacter baumannii* | Cerebrospinal Fluid |
| 60 | *Acinetobacter baumannii* | Endo Culture |
| 61 | *Acinetobacter baumannii* | Wound |
| 62 | *Acinetobacter baumannii* | Wound |
| 63 | *Acinetobacter baumannii* | Sputum |
| 64 | *Acinetobacter baumannii* | Pus |
| 65 | *Acinetobacter baumannii* | Blood Culture |
| 66 | *Acinetobacter baumannii* | Blood Culture |
| 67 | *Acinetobacter baumannii* | Pus |
| 68 | *Acinetobacter baumannii* | Pus |
| 69 | *Acinetobacter baumannii* | Urine |
| 70 | *Acinetobacter baumannii* | Sputum |
| 71 | *Acinetobacter baumannii* | Sputum |
| 72 | *Acinetobacter baumannii* | Ascites |
| 73 | *Klebsiella pneumoniae* | Sputum |
| 74 | *Acinetobacter baumannii* | Blood Culture |
| 75 | *Acinetobacter baumannii* | Pus |
| 76 | *Escherichia coli* | Urine |
| 77 | *Escherichia coli* | Urine |
| 78 | *Klebsiella pneumoniae* | Sputum |
| 79 | *Acinetobacter baumannii* | Sputum |
| 80 | *Acinetobacter baumannii* | Sputum |
| 81 | *Acinetobacter baumannii* | Sputum |
| 82 | *Acinetobacter baumannii* | Wound |
| 83 | *Klebsiella pneumoniae* | Urine |
| 84 | *Escherichia coli* | Urine |
| 85 | *Acinetobacter baumannii* | Blood Culture |
| 86 | *Acinetobacter baumannii* | Blood Culture |
| 87 | *Klebsiella pneumoniae* | Wound |
| 88 | *Klebsiella pneumoniae* | Pus |
| 89 | *Klebsiella pneumoniae* | Sputum |
| 90 | *Klebsiella pneumoniae* | Blood Culture |
| 91 | *Klebsiella pneumoniae* | Urine |
| 92 | *Klebsiella pneumoniae* | Pus |
| 93 | *Klebsiella pneumoniae* | Sputum |
| 94 | *Klebsiella pneumoniae* | Urine |
| 95 | *Klebsiella pneumoniae* | Wound |
| 96 | *Klebsiella pneumoniae* | Pus |
| 97 | *Escherichia coli* | Urine |
| 98 | *Klebsiella pneumoniae* | Blood Culture |
| 99 | *Enterobacter aerogenes* | Sputum |
| 100 | *Klebsiella pneumoniae* | Pus |
| 101 | *Klebsiella pneumoniae* | Wound |
| 102 | *Klebsiella pneumoniae* | Blood Culture |
| 103 | *Enterobacter aerogenes* | Sputum |
| 104 | *Escherichia coli* | Pus |
| 105 | *Klebsiella pneumoniae* | Pus |
| 106 | *Klebsiella pneumoniae* | Wound |
| 107 | *Klebsiella pneumoniae* | Wound |
| 108 | *Klebsiella pneumoniae* | Blood Culture |
| 109 | *Escherichia coli* | Wound |
| 110 | *Klebsiella pneumoniae* | Sputum |
| 111 | *Acinetobacter baumannii* | Blood Culture |
| 112 | *Klebsiella pneumoniae* | Pus |
| 113 | *Klebsiella pneumoniae* | Wound |
| 114 | *Escherichia coli* | Urine |
| 115 | *Klebsiella pneumoniae* | Blood Culture |
| 116 | *Klebsiella pneumoniae* | Sputum |
| 117 | *Klebsiella pneumoniae* | Blood Culture |
| 118 | *Klebsiella pneumoniae* | Blood Culture |

**Table S2: List of antibiotics used in susceptibility testing.**

| **Abbreviation** | **Antibiotic name** | **Abbreviation** | **Antibiotic name** |
| --- | --- | --- | --- |
| PI | Piperacillin | GEN | Gentamicin |
| TCC | Ticarcillin/Clavulanic acid | AZM | Azithromycin |
| CZ | Cefazolin | DO | Doxycyclin |
| AT | Aztreonam | CIP | Ciprofloxacin |
| IPM | Imipenem | COT | Cotrimoxazole |
| DOR | Doripenem | CM | Chloramphenicol |
| ETP | Ertapenem | FO | Fosfomycin |
| MRP | Meropenem | NIT | Nitrofurantoin |

**Table S3: Antibiotic resistance susceptibility of clinical Isolates.**

| Antibiotic disc | *Escherichia coli* | | | *Klebsiella pneumoniae* | | | *Acinetobacter baumannii* | | |
| --- | --- | --- | --- | --- | --- | --- | --- | --- | --- |
|  | R | I | S | R | I | S | R | I | S |
| Cefazoline | 8 | 0 | 0 | 36 | 0 | 0 | — | — | — |
| Ertapenem | 8 | 0 | 0 | 36 | 0 | 0 | — | — | — |
| Doripenem | 0 | 3 | 5 | 25 | 4 | 7 | 2 | 0 | 4 |
| Imipenem | 3 | 1 | 4 | 22 | 9 | 5 | 6 | 0 | 0 |
| Meropenem | 0 | 3 | 5 | 29 | 6 | 1 | 3 | 2 | 1 |
| Ticarcillin/ Clavulanic acid | 8 | 0 | 0 | 36 | 0 | 0 | 6 | 0 | 0 |
| Azteronam | 5 | 3 | 0 | 28 | 6 | 2 | — | — | — |
| Gentamycin | 4 | 0 | 4 | 29 | 1 | 6 | 5 | 0 | 1 |
| Azithromycin | 4 | 0 | 4 | 29 | 0 | 7 | — | — | — |
| Doxycyline | 4 | 1 | 3 | 2 | 4 | 30 | 1 | 0 | 5 |
| Ciprofloxacin | 8 | 0 | 0 | 31 | 3 | 2 | 5 | 1 | 0 |
| Fosfomycin | 0 | 0 | 8 | 3 | 0 | 33 | — | — | — |
| Nitrofurantoin | 0 | 0 | 8 | 30 | 2 | 4 | — | — | — |
| Chloramphenicol | 0 | 3 | 5 | 8 | 5 | 23 | — | — | — |
| Co-trimoxazole | 7 | 0 | 1 | 28 | 0 | 8 | 5 | 0 | 1 |
| Pipercillin | — | — | — | — | — | — | 6 | 0 | 0 |
| R=Resistant, I=Intermediate, S=Sensitive | | | | | | | | | |
